# Author Correction: Near-surface softening and healing in eastern Honshu associated with the 2011 magnitude-9 Tohoku-Oki earthquake

**DOI:** 10.1038/s41467-021-22026-1

**Published:** 2021-03-05

**Authors:** Su-Yang Wang, Hai-Yang Zhuang, Hao Zhang, Hong-Jun He, Wei-Ping Jiang, Er-Lei Yao, Bin Ruan, Yong-Xin Wu, Yu Miao

**Affiliations:** 1 School of Civil and Hydraulic Engineering, Huazhong University of Science and Technology, Wuhan, China; 2 Institute of Geotechnical Engineering, Nanjing Tech University, Nanjing, China; 3 Institute of Engineering Mechanics, China Earthquake Administration, Harbin, China; 4 Changjiang River Scientific Research Institute, Wuhan, China; 5 Hohai University, Nanjing, China

**Keywords:** Natural hazards, Seismology

Correction to: *Nature Communications* 10.1038/s41467-021-21418-7, published online 22 February 2021.

The original version of this Article omitted Su-Yang Wang and Hai-Yang Zhuang as equally contributing authors. This has now been corrected in both the PDF and HTML versions of the Article.

