# Supplementary material for: Near-surface softening and healing in eastern Honshu associated with the 2011 magnitude-9 Tohoku-Oki Earthquake
Source: Nat Commun. 2021 Feb 22;12:1215. doi: 10.1038/s41467-021-21418-7 (PMC7900222; doi:10.1038/s41467-021-21418-7)
Supplement: Supplementary file 1 — Supplementary Information [file 41467_2021_21418_MOESM1_ESM.pdf]

Extended figures and tables

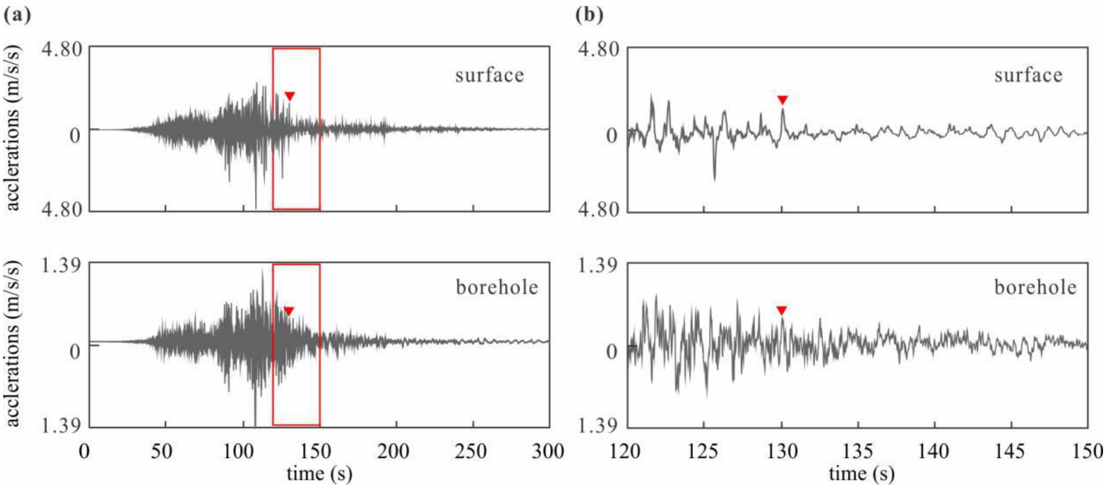

Supplementary Figure 1: (a) Horizontal acceleration seismograms of the 2011 Tohoku-Oki Earthquake at FKSH14; (b) Enlarged part of the seismograms marked with a red box in the left subplot.

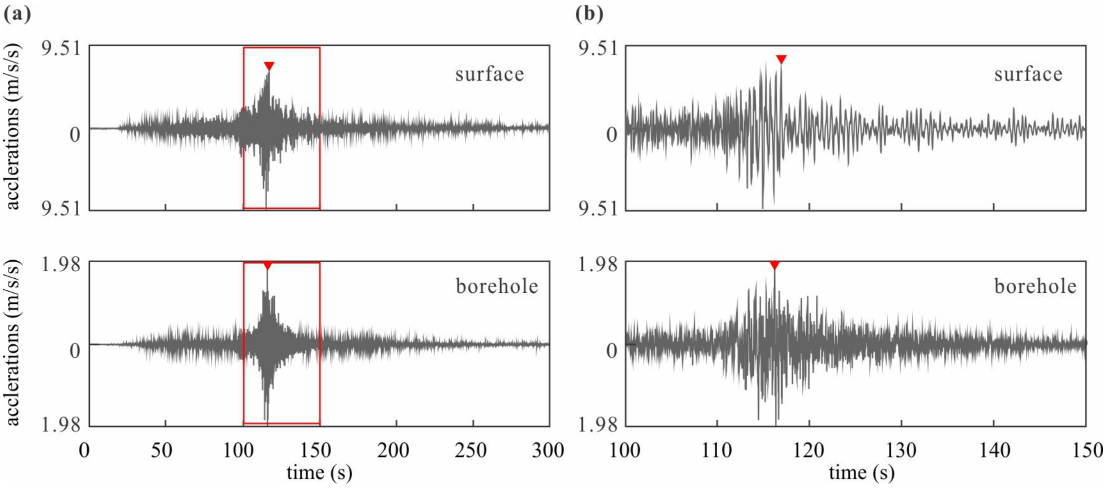

Supplementary Figure 2: (a) Horizontal acceleration seismograms of the 2011 Tohoku-Oki Earthquake at IBRH11; (b) Enlarged part of the seismograms marked with a red box in the left subplot. The abrupt drop in the waveform amplitude before and after the last significant acceleration peak of the surface seismograms indicates the possibility of incipient quasi-liquefaction at this station.

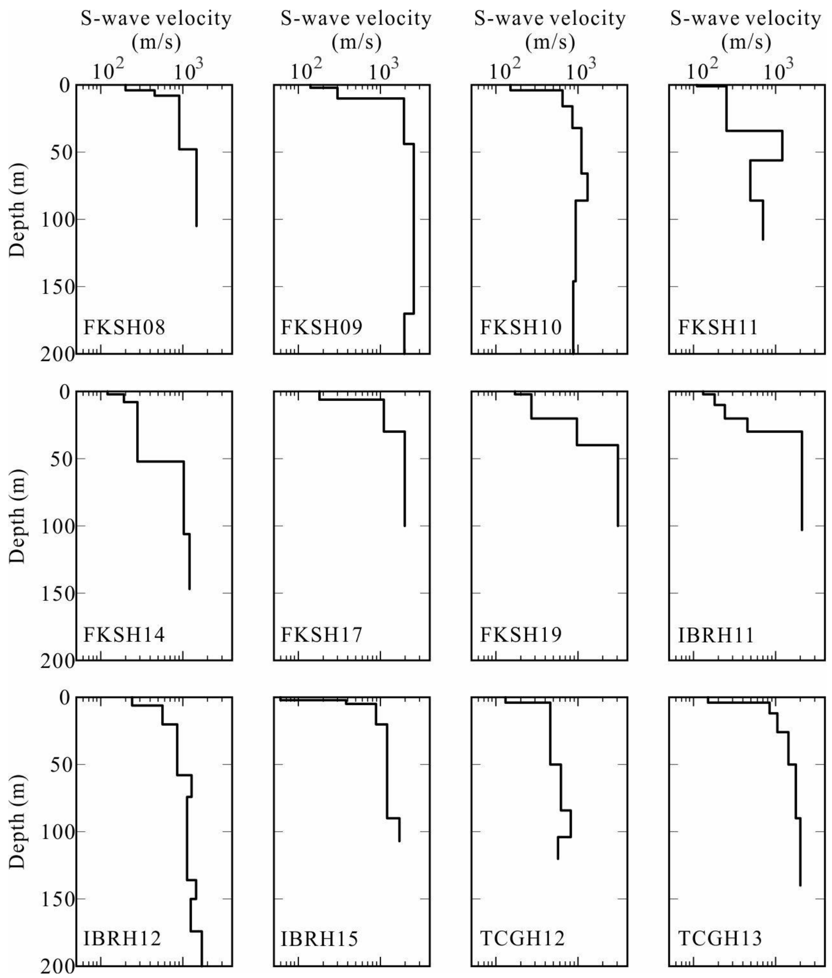

Supplementary Figure 3. Shear wave velocity profiles of the selected seismic stations from P-S logging tests.

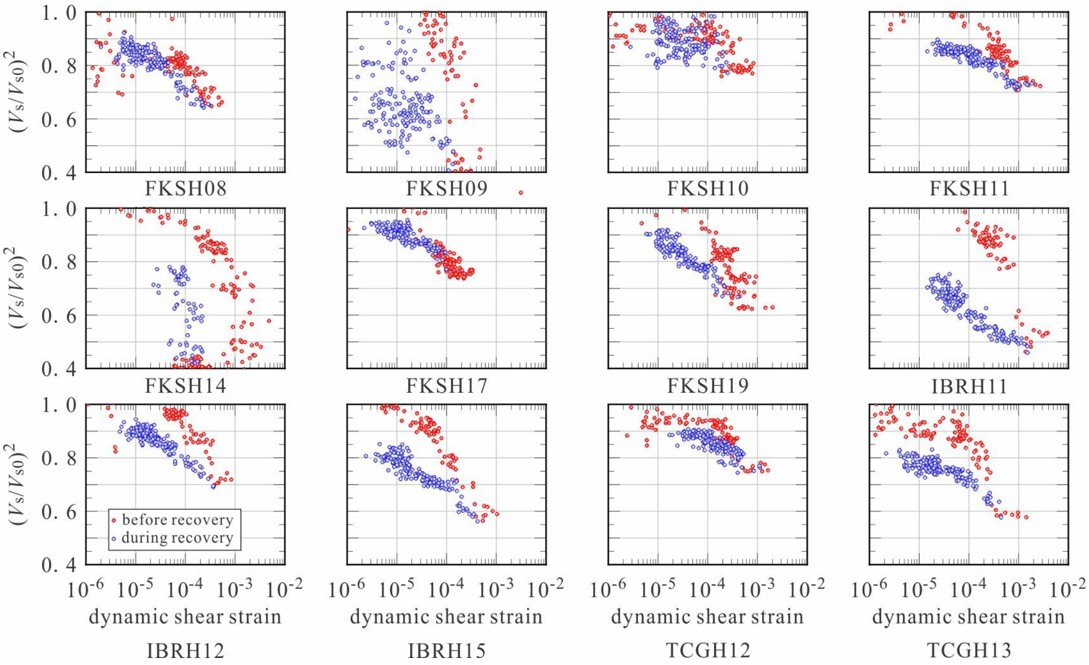

Supplementary Figure 4. Shear modulus degradation as a function of dynamic shear strain for the mainshock seismograms. The shear modulus is proportional to the square of velocity $G/G_0 \approx (Vs/Vs_0)^2$. The dynamic shear strain is calculated by v(t)/Vs30*, in which v(t) is the maximum value in each second of the velocity time histories at the surface, and Vs30* = $Vs30 \times Vs/Vs_0$ where Vs30 denotes time-averaged shear-wave velocity to a depth of 30 m. During the recovery process, there is a certain correlation between the shear modulus ratio and the dynamic strain for a majority of the stations, but the recovery of the shear modulus does not follow the original descending path, which is attributed to the combined effect of slow and fast dynamics.

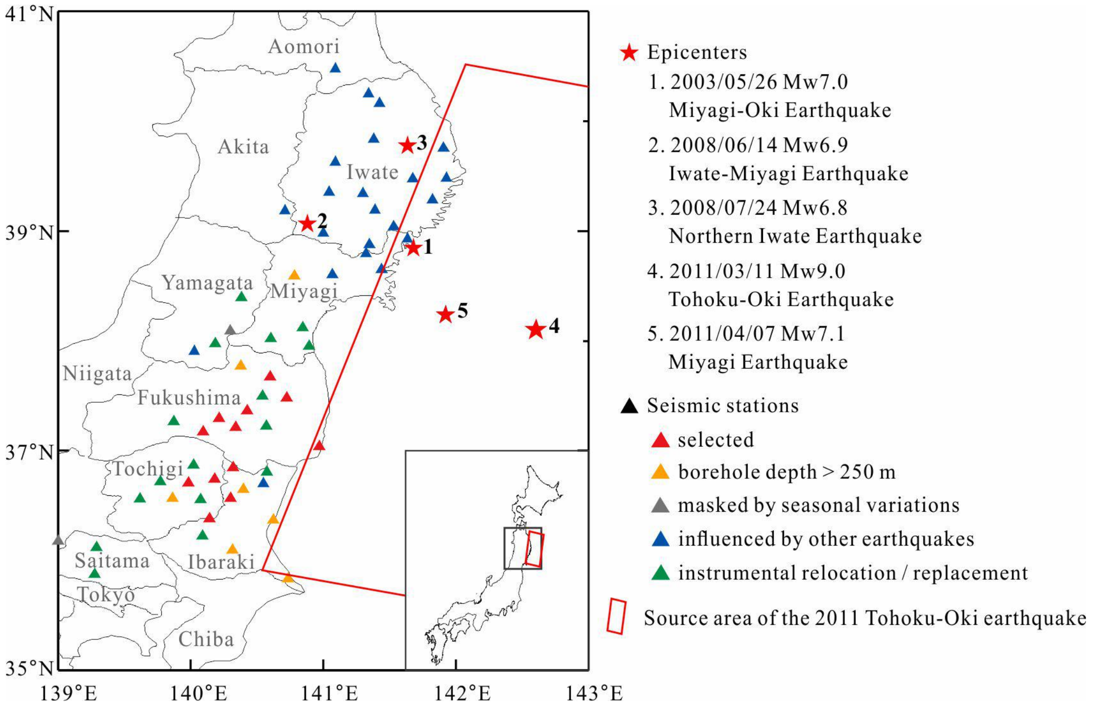

Supplementary Figure 5: Map of Northern Honshu Island, Japan, with locations of 59 KiK-Net stations whose peak ground accelerations exceeded 2 m/s^2 during the Tohoku-Oki Earthquake. The northeast corner of Honshu were struck by at least four Mw7-class earthquakes in the past two decades.

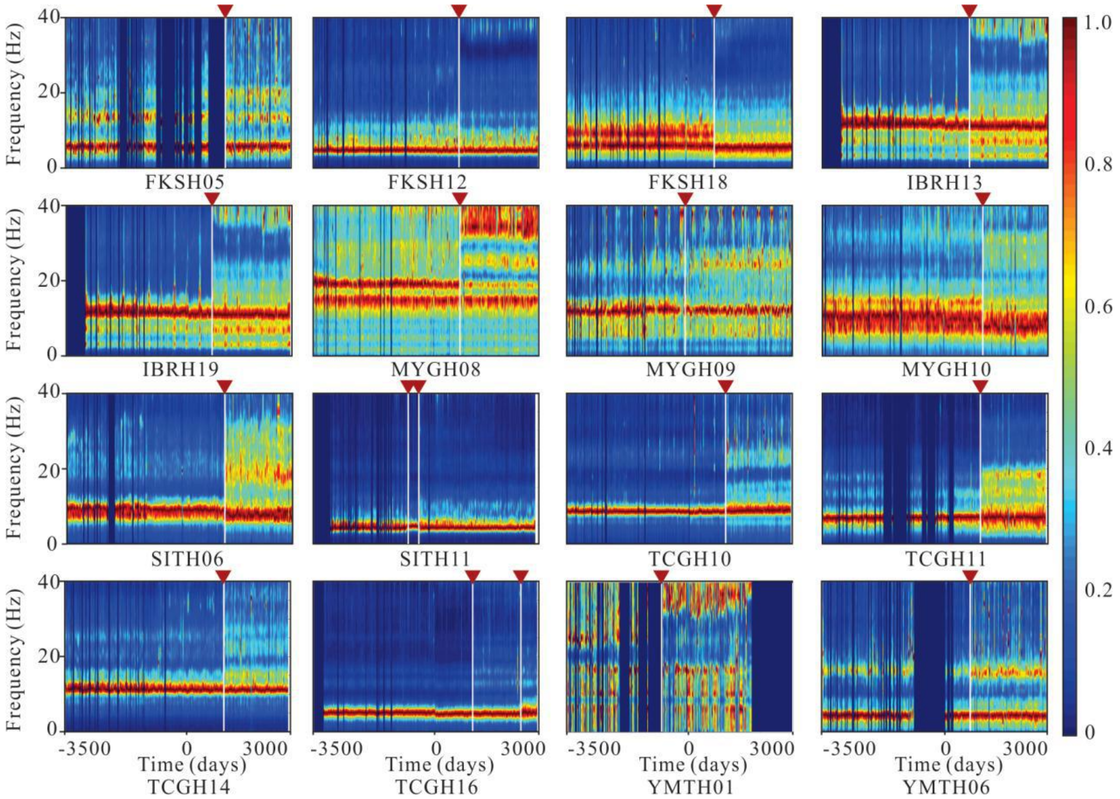

Supplementary Figure 6: Normalized surface-to-borehole spectral ratios for 16 KiK-net stations, as the evidences of instrumental replacement or relocation. The white line represents the date when the spectral ratios suddenly changed. The zero day indicates 11 March 2011.

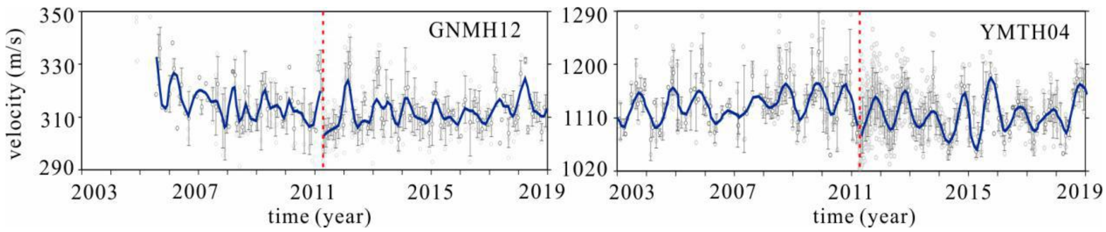

Supplementary Figure 7: Temporal variations of near-surface seismic velocity at two KiK-net stations whose velocity recovery processes are masked by strong seasonal changes. The dots and vertical error bars represent raw data and the standard deviations of monthly mean, respectively. The blue line shows smoothed trend line using LOWESS (locally weighted scatter-plot smoothing) method with a smoothing factor of 0.1, and the red vertical line indicates the origin time of the Tohoku-Oki Earthquake.

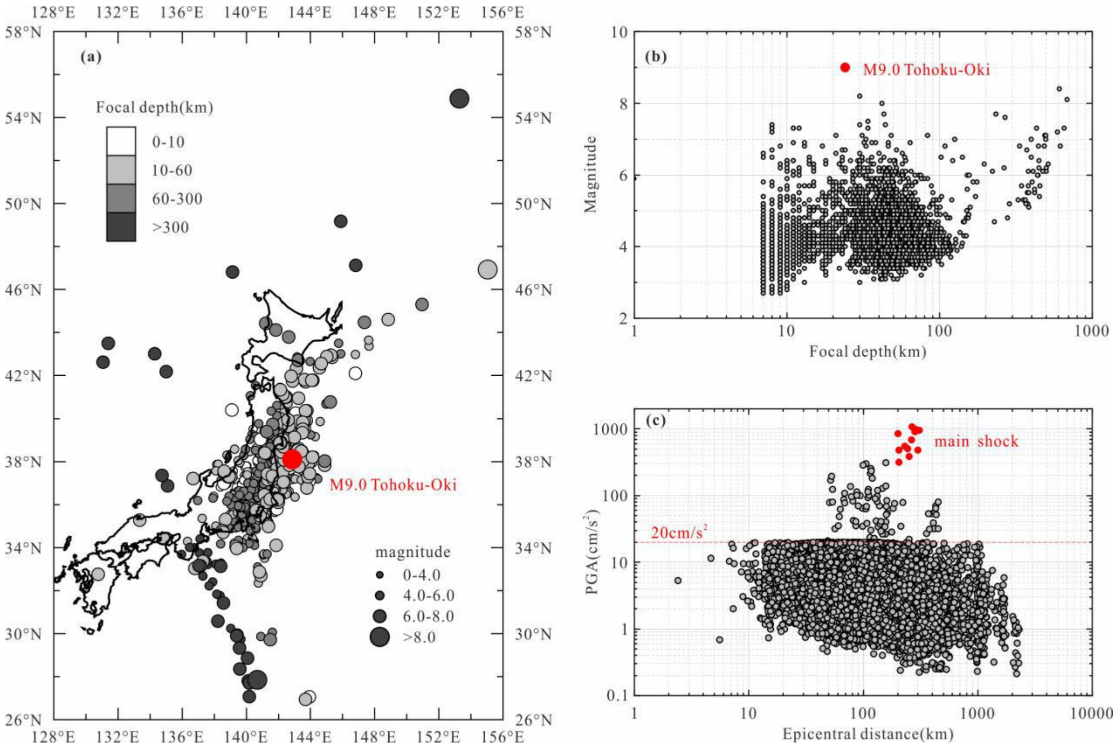

Supplementary Figure 8: (a) Distribution of the epicenters of the selected earthquakes; (b) Magnitude and focal depth of the selected earthquakes; (c) PGA and epicentral distance of the selected records.

Supplementary Table 1: Details of the recovery process for the selected stations

Site Code	First Stage			Second Stage			α_1/α_2	$\Delta c_1/\Delta c_2$
	slope (α_1)	intercept	R-square	slope (α_2)	intercept	R-square		
FKSH08	0.068	-0.220	0.974	0.013	-0.095	0.947	5.21	2.02
FKSH09	0.114	-0.398	0.973	0.029	-0.239	0.930	4.00	0.85
FKSH10	0.025	-0.100	0.989	0.009	-0.068	0.884	2.67	1.49
FKSH11	0.047	-0.180	0.933	0.011	-0.097	0.955	4.50	1.13
FKSH14	0.119	-0.389	0.916	0.012	-0.116	0.949	9.90	2.86
FKSH17	0.038	-0.118	0.993	0.005	-0.047	0.744	7.42	2.26
FKSH19	0.062	-0.203	0.978	0.009	-0.087	0.909	6.97	1.70
IBRH11	0.127	-0.446	0.967	0.017	-0.151	0.938	7.55	2.00
IBRH12	0.057	-0.175	0.983	0.008	-0.074	0.959	7.18	1.98
IBRH15	0.072	-0.268	0.977	0.017	-0.142	0.978	4.31	1.41
TCGH12	0.039	-0.148	0.975	0.007	-0.068	0.972	5.67	1.79
TCGH13	0.066	-0.252	0.989	0.017	-0.145	0.988	3.84	1.22

α_1 and α_2 are the log slopes in the first and the second stages, respectively; Δc_1 and Δc_2 are the recovery amounts of the shear-wave velocity during the first and the second stages, respectively.

Supplementary Table 2: Details of the coseismic velocity variation for the selected stations

Site Code	PGA (m/s^2)	MVR (%)	PGV (m/s)	Vs30 (m/s)	MDS	SVS
FKSH08	3.87	19.88	0.2513	562.5	$4.47x10^{-4}$	445
FKSH09	5.52	37.40	0.2272	584.6	$3.89x10^{-4}$	962
FKSH10	10.75	11.92	0.3624	487.0	$7.44x10^{-4}$	160
FKSH11	5.03	15.71	0.5794	239.8	$2.42x10^{-3}$	65
FKSH14	4.80	46.29	0.8621	236.6	$3.64x10^{-3}$	127
FKSH17	3.18	14.10	0.2115	544.0	$3.89x10^{-4}$	363
FKSH19	8.57	21.10	0.5795	338.1	$1.71x10^{-3}$	123
IBRH11	9.51	32.24	0.6762	242.5	$2.79x10^{-3}$	116
IBRH12	6.83	16.88	0.3445	485.7	$7.09x10^{-4}$	238
IBRH15	9.87	24.94	0.3620	450.4	$8.04x10^{-4}$	310
TCGH12	4.81	13.71	0.5210	343.7	$1.52x10^{-3}$	90
TCGH13	9.06	24.00	0.6263	573.6	$1.09x10^{-3}$	220

The PGA and PGV represent peak ground acceleration and peak ground velocity, respectively. The MVR and MDS denote maximum velocity reduction and maximum dynamic strain, respectively. The Vs30 and SVS are time-averaged shear-wave velocity to a depth of 30 m and seismic velocity susceptibility, respectively. MDS = PGV/Vs30, and SVS = MVR/MDS.

Supplementary Table 3: Details of the select seismic stations

Site Code	Prefecture	Latitude	Longitude	Altitude (m)	Depth (m)	Number of Records	
						Selected	Total
FKSH08	Fukushima	37.2822	140.2144	343	105	1244	1402
FKSH09	Fukushima	37.3530	140.4264	260	200	2134	2470
FKSH10	Fukushima	37.1616	140.0930	565	200	2273	2728
FKSH11	Fukushima	37.2006	140.3386	286	115	1654	1934
FKSH14	Fukushima	37.0264	140.9702	3	147	2045	2324
FKSH17	Fukushima	37.6636	140.5974	205	100	1832	2007
FKSH19	Fukushima	37.4703	140.7227	510	100	2051	2439
IBRH11	Ibaraki	36.3701	140.1401	67	103	1995	2581
IBRH12	Ibaraki	36.8369	140.3181	210	200	1971	2473
IBRH15	Ibaraki	36.5566	140.3013	45	107	2017	2489
TCGH12	Tochigi	36.6959	139.9842	162	120	2375	2764
TCGH13	Tochigi	36.7342	140.1781	135	140	1715	2163

The altitude and depth represent elevation above the sea level and borehole depth, respectively.
